# Production and Cytotoxicity of Extracellular Insoluble and Droplets of Soluble Melanin by *Streptomyces lusitanus* DMZ-3

**DOI:** 10.1155/2014/306895

**Published:** 2014-04-16

**Authors:** D. N. Madhusudhan, Bi Bi Zainab Mazhari, Syed G. Dastager, Dayanand Agsar

**Affiliations:** ^1^A-DBT Research Laboratory, Department of Microbiology, Gulbarga University, Gulbarga 585 106, India; ^2^NCIM Resource Centre, Biochemical Sciences Division, National Chemical Laboratory (CSIR), Pune 411008, India

## Abstract

A *Streptomyces lusitanus* DMZ-3 strain with potential to synthesize both insoluble and soluble melanins was detected. Melanins are quite distinguished based on their solubility for varied biotechnological applications. The present investigation reveals the enhanced production of insoluble and soluble melanins in tyrosine medium by a single culture. *Streptomyces lusitanus* DMZ-3 was characterized by 16S rRNA gene analysis. An enhanced production of 5.29 g/L insoluble melanin was achieved in a submerged bioprocess following response surface methodology. Combined interactive effect of temperature (50°C), pH (8.5), tyrosine (2.0 g/L), and beef extract (0.5 g/L) were found to be critical variables for enhanced production in central composite design analysis. An optimized indigenous slant culture system was an innovative approach for the successful production (264 mg/L) of pure soluble melanin from the droplets formed on the surface of the culture. Both insoluble and soluble melanins were confirmed and characterized by Chemical, reactions, UV, FTIR, and TLC analysis. First time, cytotoxic study of melanin using brine shrimps was reported. Maximum cytotoxic activity of soluble melanin was Lc_50_-0.40 µg/mL and insoluble melanin was Lc_50_-0.80 µg/mL.

## 1. Introduction

Melanin, a polyphenolic polymer formed by the oxidative polymerization of phenolic and/or indolic compounds, was generally produced from the oxidation of L-tyrosine by tyrosinase or laccase to L-DOPA and dopaquinones, finally to dihydroxyindole carboxylic acid and their reduced forms [1]. Melanins are commonly found in animals, plants, bacteria, and fungi [2]. In humans, they are found mainly in the skin and hair as dark colored pigments. In bacteria and fungi, melanins are found in their cell wall. The biological melanins are commonly known based on the color and the substrate from which they originate. Eumelanin is blackish brown, Pheomelanin is yellow to red, and Pyomelanin is brown in color [3]. Eumelanin is the predominant pigment synthesized in humans and microorganisms, especially in bacteria and fungi [2].

Melanin is commercially extracted from cuttlefish and depends on irregular supply of natural material and also is expensive [4]. Plenty of literature is available regarding the synthesis and production of eumelanin by different bacteria [5, 6] and fungi [7, 8]. The production of melanin by recombinant* E. coli* under optimized submerged bioprocess was reported by Muñoz et al. [9]. Dastager et al. [10] and Quadri and Agsar [11] have reported the production of melanin by Streptomyces species. Manivasagan et al. [12] and Surwase et al. [13] have reported the production of melanin by* Actinoalloteichus sp.* and* Brevundimonas sp.*, respectively, employing Response Surface Method (RSM). RSM using different statistical designs is an important approach to optimize the process condition for the enhanced production of bioactive molecules [14]. Formation of droplets on the surface of the colonies of few sporulated microorganisms constituting mainly enzymes or antibiotics or pigments was reported [15–17].

Melanin plays an important role in humans and its lack leads to several abnormalities and diseases. The reduced melanin in neurons causes Parkinson's disease [18]. Melanin also plays an important role in microorganisms against damages from high temperatures, chemical stress, and biochemical threats [19]. The role of biologically active melanin includes being cytotoxic, antitumor [20], antivenin [21], antiviral [22], and radio protective [23]. Sun-screens containing water soluble melanin protect against harmful UV radiations. Water soluble melanins are used in solid plastic films, lenses, paints, varnishes, and other surface protection formulations to provide greater UV protections [24]. AIDS treatment news [25] reveals the selective antiviral activity of synthetic soluble melanin against human immunodeficiency virus [22]. However, it is critical for melanin to be water soluble for a better commercial potential in biotechnological applications. Insoluble melanins require sever treatments such as boiling in strong alkali or the use of strong oxidants for making them water soluble, which often damages them [26]. The present investigation was undertaken to produce insoluble melanin and soluble melanin from droplets of* Streptomyces*, as no literature is available regarding this approach. The standardization of production of bioactive molecules from such droplets is a novel criterion explaining production of melanin by* Streptomyces* in unique form. Further, the cytotoxic activity of melanins was evaluated using brine shrimps. The brine shrimp cytotoxic activity has been found out as safe, practical, and economical to determine the bioactivity of the synthetic compounds [27], which showed a significant correlation with* in vitro* growth check for human solid tumor cell lines [28].

## 2. Materials and Methods

### 2.1. Screening of* Streptomyces*

*Streptomyces* collection preserved in our A-DBT (Actinomycetes-Diversity and Bioprocess Technology) research laboratory was screened by plate culture for the synthesis of melanin on starch tyrosine agar (STA): Starch 10 g, K_2_HPO_4_ 2 g, KNO_3_ 2 g, NaCl 2 g, Tyrosine 4 g, MgSO_4_ 0.05 g, CaCO_3_ 0.3 g, FeSO_4_ 0.01 g, Agar 20 g, deionized water 1 L, and pH 8.0 and also on tyrosine agar (TA): Gelatin 5 g, Tyrosine 5 g, beef extract 3 g, Agar 20 g, deionized water 1 L, and pH 8.0 [29]. The plates inoculated with test isolates were incubated at 40°C for 120 h and observed for synthesis of melanin based on the intensity of dark brown pigmentation and degree of zone of catalysis as reported by Nicolaus et al. [3] and Shivaveerakumar et al. [30].

### 2.2. Molecular Characterization of* Streptomyces*

Efficient isolate for melanin synthesis was characterized by 16S rRNA analysis [31]. Genomic DNA was prepared by using Chelex-100 (Sigma-Aldrich, USA) chelating ion exchange resin method [32]. Employing about 100 nanogram DNA, 16S rRNA amplified using universal F27 (5′AGAGTTTGATCMTGGCTCAG-) and R1525 (5′TACGG(C/T) TACCTTGTTACGACTT) primers. Accuracy of PCR product was visualized on agarose gel and sequenced using a BigDye Terminator kit, version 3.1, on an automatic ABI 3100 sequencer (Applied Biosystems Inc.). The sequences obtained were analyzed using NCBI Blast search and EzTaxon [33] to restore closest relatives and phylogenetic tree was obtained.

### 2.3. Production of Insoluble Melanin

Submerged bioprocess in tyrosine broth was standardized for the production of melanin using* Streptomyces lusitanus* DMZ-3, employing important process variables one at a time and keeping others at a constant level [34]. pH (7.0, 7.5, 8.0 and 8.5) of the medium, inoculum size (from 1 × 10^6^ to 1 × 10^9^ with interval of 1 × 10^1^), incubation temperature (35, 40, 45, 50 and 55°C), period of incubation (48, 72, 96, 120, 144 and 168 h), and rate of agitation (120, 140, 160, 180 and 200 rpm) were manually optimized. The influence of various carbon sources (Starch, glucose, sucrose, maltose, and beef extract at 0.2 to 2.0% concentrations), nitrogen sources (soyabean meal, ammonium nitrate, casein, and tyrosine at 0.2 to 2.0% concentrations), and mineral salts (CuSO_4_, MgSO_4_, FeSO_4_, MnSO_4_, and K_2_HPO_4_ at 0.05 to 0.25% concentrations) were also optimized.

Response Surface Method (RSM) with central composite design (CCD) was employed [12] to resolve the optimum combination and interactive effect of critical process variables on the enhanced production of melanin. The CCD of 30 runs was set using the Design Expert Software, USA (Version 7.0). All the experiments were carried out in duplicate and average of melanin production obtained was considered as the dependent variables or responses (*Y*). The predicted response was calculated from the second degree polynomial equation, which included all the terms. *Y* = *β*_0_ + ∑*β*_*i*_ *X*_*i*_ + ∑*β*_*ii*_ *X*_*i*_^2^ + ∑*β*_*ij*_ *X*_*i*_ *X*_*j*_, where *Y* stands for the response variable, *β*_0_ is the intercept coefficient, *β*_*i*_ represents the coefficient of the linear effect, *β*_*ii*_ the coefficient of quadratic effect, and *β*_*ij*_ the *ij*th interaction coefficient effect. *X*_*i*_ *X*_*j*_ are input variables which influence the response variable *Y* and *β*_*i*_ is the *i*th linear coefficient [35]. Other parameters which have no much role in production of melanin were kept constant. The statistical and numerical analysis of the model was performed with the analysis of variance (ANOVA). The statistical significance of the model was analyzed by the Fisher's *F*-test, its associated probability *P*(*F*), correlation coefficient *R*, and determination coefficient *R* ^2^, which explains the quality of polynomial model. The quadratic models were represented as contour plots (three-dimensional) and response surface curves were created for each variable. The model was validated for enhanced production of melanin, at specific level of optimized critical process variables.

The extraction and purification of the melanin were carried out as per the standard protocols described by Fava et al. [36] and Harki et al. [37], respectively. The incubated broth was centrifuged at 8,000 g for 15 minute to separate the cell mass and the pigment. Extracted dried pigment pellet was subjected to the dialysis in cellulose membrane against phosphate buffer of pH 7.0 and purified by column using Silica Gel material of 60–120 mesh size.

### 2.4. Production of Soluble Melanin

An indigenous method of slant culture system was standardized and operated for the synthesis and extraction of soluble pigment. Potential isolate of* Streptomyces* was inoculated on the slants of tyrosine agar. The 50 mL capacity borosilicate glass tube with 15 mL medium was employed for the synthesis of melanin formed as clearly visible pigment droplets on the surface of the slant culture. The slants inoculated were incubated at 45°C for 120 h. The dark brown pigment droplets present on the entire surface of the culture were completely extracted using micropipette and dried in hot air oven at 60°C for 1 h. Replicates of three slants were considered to calculate a simple arithmetic mean of total soluble melanin produced per liter of tyrosine medium.

### 2.5. Confirmation of Melanin

The pigments obtained by both submerged bioprocess and slant culture were confirmed as insoluble and soluble melanin by following chemical method, UV-vis spectroscopy, FT-IR spectroscopy, and thin layer chromatographic techniques. The solubility of pigments in deionized water, 1 N HCl, 1 N NaOH, 1 N KOH, 1 N NH_4_OH, ethanol, acetone, chloroform, and benzene was assessed [1, 38]. The reaction of the pigment with oxidizing agent H_2_O_2_ (30%), reducing agents H_2_S, and sodium hydrosulfite (5%) was observed and recorded for the confirmation of the pigment as a melanin. The pigment was also subjected to precipitation reaction with FeCl_3_ (1%), ammonical silver nitrate, and potassium ferricynide. UV-visible absorption spectrum in the region of 200 to 600 nm was observed [39] for a characteristic property of a melanin using Systronics 2201 double beam UV-visible spectrophotometer. The pigments were directly subjected to FT-IR Spectroscopy analysis and spectrum was recoded at 4000 to 500 cm^−1^ [40] using thermo Nicolet iS5 FT-IR Spectroscopy. The confirmation of the pigment as melanin was also performed by thin layer chromatography [41]. The pigment extracts were separated and compared with standard melanin using silica gel chromatography plate (Merck TLC Silica Gel 60 F_254_). The separation was made using the different proportions of organic solvents such as chloroform, hexane, butanol, acetic acid, and methanol. After optimizing the solvent proportions, separate bands were observed staining with iodine.

### 2.6. Cytotoxic Activity of Melanin

The cytotoxic activities of insoluble and soluble melanins were determined by following the standard protocol of Meyer et al. [42] using brine shrimps (*Artemia salina*). Artificial sea/saline water was prepared, dissolving 20 g of NaCl per liter and pH was adjusted to 8.5 with 0.1 M Na_2_CO_3_. 1 g eggs of brine shrimp was added to the 1 L seawater and incubated at 28°C for 48 h with constant air supply and light. The hatched brine shrimps were collected and rinsed in fresh seawater. The insoluble and soluble melanin concentrations (0, 1, 2, 4, 8, 16, 32, and 64 *μ*g/mL) were diluted in 5 mL seawater in separate tubes and incubated at 28°C. Sample with zero concentration of melanins was considered as control. The mortality number of brine shrimps for every 6 h up to 24 h was recorded. The percentage of mortality and lethal concentration value (LC_50_-*μ*g/mL) of melanins were calculated. The mortality end point of the bioassay was referred to as the absence of controlled forward motion during 30 seconds of observation and the concentration that killed 50% of brine shrimps as LC_50_. Criterion of toxicity for fractions was categorized as nontoxic (LC_50_ values > 1000 *μ*g/mL), poor toxic (500–1000 *μ*g/mL), and toxic (<500 *μ*g/mL) according to Déciga-Campos et al. [43].

## 3. Results and Discussion

### 3.1. Screening and Characterization of* Streptomyces*

**Figure 1:**
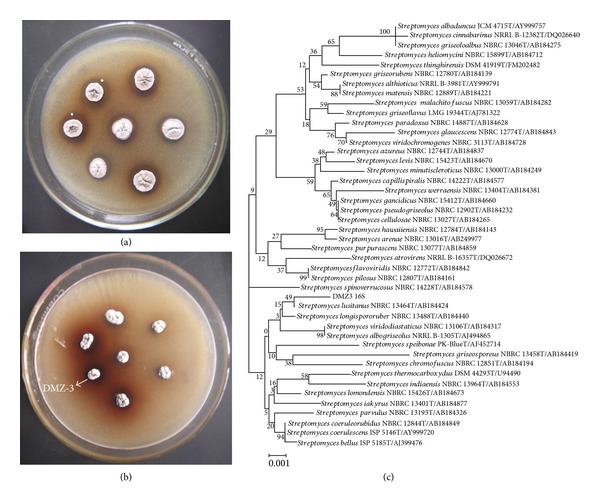
Screening of isolates for the synthesis of melanin on starch tyrosine (a), tyrosine agar (b), and phylogenetic tree (c) of* Streptomyces* DMZ-3.

Seven isolates of* Streptomyces* were screened for the extracellular synthesis of melanin on starch tyrosine and tyrosine agar. Starch casein agar is a medium prescribed by Küster and Williams [44] for the isolation of actinobacteria. Casein was replaced by tyrosine and used for the isolation and screening of* Streptomyces* for the synthesis of melanin. Degree of coloration or intensity of color (brown) was conventional method prescribed [45] for the detection of melanin synthesis by microorganisms. A varied degree of synthesis of melanin by the test isolates of* Streptomyces* was clear from the visible (Figure 1(a)) brown pigment (melanin) intensity on starch tyrosine agar. In addition to the intensity of brown pigmentation, the formation of catalytic zone around the colonies of the test isolates on tyrosine agar (Figure 1(b)) significantly reveals the synthesis of melanin. However, tyrosine agar was reported earlier [29] to be used for the differentiation of* Streptomycetes* but never recorded for the formation of catalytic zone indicating the melanin activity. Surprisingly, this medium exhibited both, high intensity of color and also catalytic zone (a clear zone around the colony catalyzing the tyrosine), indicating the synthesis of melanin. The degrees of catalytic zone developed can be considered as an innovative criterion for the selection of potential isolate targeting the production of melanin. An isolate* Streptomyces* DMZ-3 was selected as physiologically efficient and potential isolates (Figure 1(b)) for the maximum synthesis of melanin. A similar approach was reported by Shivaveerakumar et al. [30] as a novel criterion for the screening of actinobacteria aiming at the synthesis of extracellular tyrosinase.

Actinobacteria can be analyzed at various molecular levels to gain information suitable for constructing databases and effective identification. Sequence analysis of various genes provides a stable classification and accurate identification, which has become the cornerstone of modern phylogenetic taxonomy [46]. The region of 16S rRNA gene is highly variable and differs significantly between species where other areas are more conserved and suitable for identification at the generic level [47]. An almost complete 16S rRNA gene sequence of isolate DMZ-3 (1,401 nucleotides) was determined (Genbank, NCBI Accession number: KF486519). A phylogenetic tree was constructed based on 16S rRNA gene sequences to show the comparative relationship between isolate DMZ-3 and other related* Streptomyces* species (Figure 1(c)). The comparative analysis of 16S rRNA gene sequence and phylogenetic relationship reveals that isolate DMZ-3 lies in a subclade with* Streptomyces lusitanus, *sharing 99.7% of 16S rRNA gene sequence similarity.

### 3.2. Production of Insoluble and Soluble Melanin

Operation of process variables using manually one at a time and keeping others constant is a precondition to detect critical variables for the production of melanin employing response surface methodology. 4.2 g/L melanin was produced by* Streptomyces lusitanus* DMZ-3 in tyrosine medium under the optimized submerged bioprocess. Among all the optimized process variables, temperature, pH, tyrosine, and beef extract were identified as critical variables based on the level of the melanin production. Determination of important physiochemical and nutritional factors is an important criterion for the maximum production of melanin, in the development of a suitable bioprocess. Few reports are available regarding the production of extracellular and intracellular melanins. Santos and Stephanopoulos [5] and Muñoz et al. [9] reported 375 mg/L and 6 g/L of extracellular melanin production, respectively, by recombinant* E. coli*. Sajjan et al. [6] and Youngchim et al. [48] have reported the production of intracellular melanin by* Klebsiella sp*. and* Aspergillus fumigatus, *respectively. Dastager et al. [10] revealed the production of extracellular melanin by* Streptomyces* in synthetic media.

**Figure 2:**
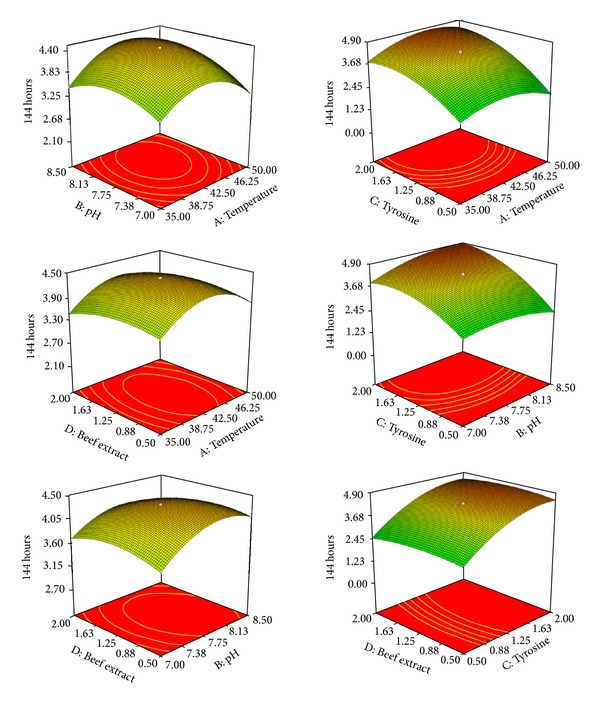
Contour plots and response surface curves of the production of insoluble melanin by* Streptomyces lusitanus.*

In the recent past, Response Surface Method, a software based statistical design, is the most validated method for the production of biomolecules, especially in the submerged fermentation. In the present investigation, an attempt was made to achieve the higher production of melanin by* Streptomyces lusitanus* DMZ-3 in submerged bioprocess. Actual and predicted values of the degree of melanin production with identified critical process variables employing RSM with CCD were shown in Table 1. Maximum response with 5.29 g/L of melanin was achieved at 9th run against a predicted value of 4.65 g/L. A polynomial equation regarding the production of melanin (*Y*) based on the regression analysis was as follows:

(1) Melanin  production(Y)=4.35+0.094X1+0.17X2+0.95X3−0.050X4−0.57X12−0.34X22−0.58X32−0.19X42+0.15X1X2+0.24X1X3+0.024X1X4+0.32X2X3−0.073X2X4+0.041X3X4,

where *X* was the response variables for melanin production with *X*_1_, *X*_2_, *X*_3_, and *X*_4_ as coded values of temperature, pH, tyrosine, and beef extract, respectively. The model characteristic response for the production of melanin was statistically analyzed (Table 2) using ANOVA. The model showed a high coefficient *R* ^2^ value of 0.8832 where standard should be >0.75 and between 0 and 1. The model *F* value of 8.10 implies the model as significant and the lack of fit *F* value was 5.62 indicating the lack of fit was not significant in relation to the pure error. The ratio greater than 4 is desirable to confirm the model as acceptable and the obtained ratio was 10.462. This revealed that the model can be used to navigate the design space. The response variables *C*, *A* ^2^, *B* ^2^, and *C* ^2^ were found to be as significant model terms. Each critical variable in the model with respect to incubation time was presented as response surface curves by contour plots (Figure 2). Every critical variable showed maximum melanin production at a constant middle level of the other variables. However, increase in the production of melanin was observed with increase in these variables. The validation of the statistical model and regression analysis considering *X*_1_(50°C), *X*_2_(8.5 pH), *X*_3_(2%), and *X*_4_(0.5%) values were evident that the use of RSM with CCD can be effectively used and the conditions are ideal for the production of melanin.

Temperature, pH, tyrosine, and beef extract were the most critical factors to produce enhanced level (5.29 g/L) melanin by* Streptomyces lusitanus* DMZ-3 in submerged bioprocess. Manivasagan et al. [12] reported the production (85.37 *μ*g/L) of melanin from* Actinoalloteichus sp.* MA-32, with glycerol, L-tyrosine, NaCl, and trace salt solution as critical process variables employing response surface methodology with central composite design. However, Surwase et al. [13] revealed pH, tryptone, L-tyrosine, and copper sulphate as critical variables for the production (6.8 g/L) of melanin by a bacterium,* Brevundimonas sp*. SGJ employing response surface method with Box-Behnken method. It is evident from the present investigation and with reported literature that a critical process variable, either physicochemical or nutritional, does vary from one organism to another, irrespective of method being employed for the production of melanin. The physiological and metabolic nature of the organism involved might regulate the process variables required for the production of melanin in given bioprocess. The simple ingredients of the medium for an efficient production of melanin are the added advantage of the present investigation.

**Figure 3:**
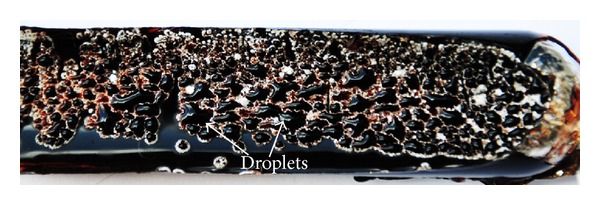
Formation of dark brown pigment droplets on the surface of the slant culture of* Streptomyces lusitanus *DMZ-3.

The preserved culture of* Streptomyces lusitanus* DMZ-3 on tyrosine agar, to our surprise, could show the formation of dark brown pigment droplets (Figure 3) on the entire surface of the slant culture. This natural phenomenon of synthesis of dark brown pigment in the form of droplets was explored and standardized for the production of melanin. It is a novel approach to progress the production of melanin employing a slant culture system. An entire slant culture grown on 15 mL medium could generate about 1 mL droplet and upon drying 3.96 mg of pigment was obtained. A simple experiment, designed statistically in triplicate, revealed the production of 264 mg pigment per liter of tyrosine medium by* Streptomyces lusitanus* DMZ-3. Early literature reports the formation of droplets on the surface of the colonies of fungi [17] and* Streptomyces* [49, 50], consisting broad range of enzymes, antibiotics, and pigments. The present investigation reveals the formations of droplets on the surface of* Streptomyces* lusitanus and for the first time droplets were reported to contain exclusively pure soluble melanin. It is interesting to note that the formation of droplets occurred only when the isolate was grown at 45°C on tyrosine agar with a pH of 8.0. It confirms the earlier reports [51] indicating the formation of droplets by organisms under stress conditions. Martín et al. [52] reported that the gene for the secretion of this kind of metabolite droplets lies within the gene cluster for the corresponding biosynthesis. In depth investigations are essential to understand specific genes regulating the synthesis of soluble melanin droplets by* Streptomyces lusitanus *DMZ-3. A water soluble melanin has been reported [53] in a mutant strain of* Bacillus thuringiensis.* However, few patents reveal the production of water soluble synthetic [54] and fungal melanin [55].

### 3.3. Confirmation and Characterization of Melanins

The pigment obtained under submerged bioprocess was purified by chromatographic technique using silica gel columns. Thus purified pigment (pigment 1) and the pigment directly extracted from the slant culture system (pigment 2) were analyzed for their characteristic chemical features (Table 3). Interestingly, both pigments 1 and 2 produced by the same organism* Streptomyces lusitanus* DMZ-3 were revealed to be insoluble and soluble in water, respectively. However, solubility of both pigments in other alkaline solutions and organic solvents is one and the same. The other chemical reactions of both the pigments were also similar. In all, blackish brown color and total chemical reactions confirm [1, 56] pigments 1 and 2 as insoluble and soluble melanin, respectively.

**Figure 4:**
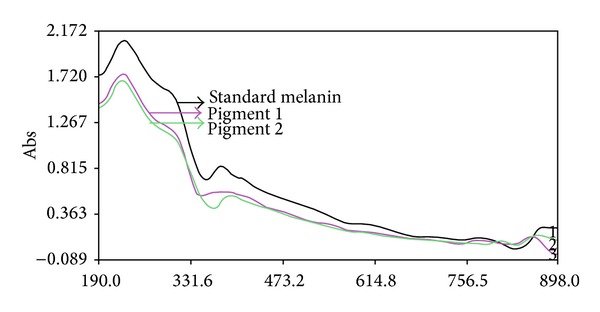
UV-visible spectrophotometric analysis of insoluble and soluble melanins.

**Figure 5:**
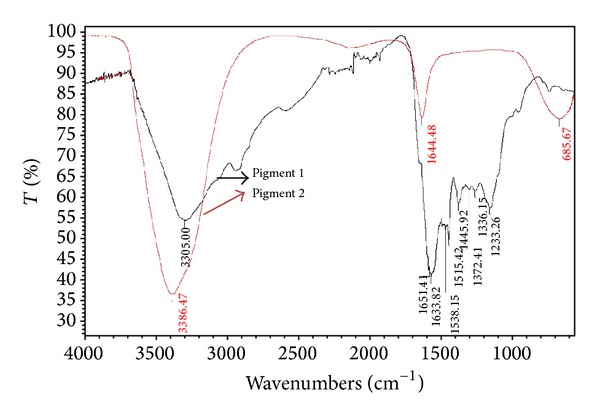
FT-IR Spectroscopic analysis of pigments 1 and 2.

**Figure 6:**
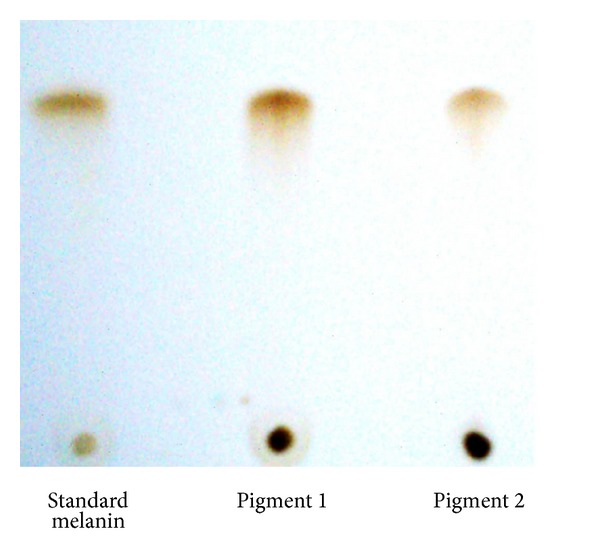
Thin layer chromatograph of insoluble and soluble melanins.

Both pigments gave maximum UV absorption between 200 and 300 nm but decreased towards visible range in the similar line of standard melanin under UV-Vis spectrophotometer (Figure 4), revealing [57] the typical nature of the melanin absorbance. The FT-IR spectrophotometric (Figure 5) analysis of insoluble and soluble pigments showed the absorption at 3305.00 cm^−1^ (–OH and –NH bonds), 1651.41 cm^−1^ (aromatic stretch C=C), and 3386.47 cm^−1^ and 1644.48 cm^−1^ respectively. The absorbance at these ranges proves [58, 59] the pigments as melanins and the peaks in the soluble melanin indicate the purity. Finally, the pigments were separated after standardizing the solvent proportion with 1 : 4 : 1 ratio of 0.1 N NaOH, ethanol, and chloroform, respectively. The separated bands (Figure 6) reveal the equal *R*_*f*_ with standard melanin.

### 3.4. Cytotoxicity of Melanins

Assessment of cytotoxicity of chemicals using cell lines is not an uncommon procedure and is accurately correlated [60] with the assessment of cytotoxicity using brine shrimps. The brine shrimp assay method is considered as an excellent alternate option to assess the cytotoxic activity of the biological product [61]. From the beginning of its introduction to standardization [42], this* in vivo* test had successfully been adopted for the bioassay of active cytotoxic and antitumor agents [62]. Further the lethal concentration of brine shrimp can be correlated with the lethal dose in mice and was explained using medicinal plants earlier [63]. The number of brine shrimps survived and the percentage of mortality for 6 h, 12 h, and 24 h was summarized in Table 4. After 24 h, the total mortality was 100% in the highest concentration (64 *μ*g/mL) of soluble melanin and 95% mortality was observed in insoluble melanin. The LC_50_ value was 0.40 *μ*g/mL for soluble melanin and 0.80 *μ*g/mL for insoluble melanin. Both pigments exhibit higher cytotoxic activity as LC_50_ value of both showed less than 500 *μ*g/mL [43]. However, soluble melanin could reveal 100% mortality and greater cytotoxic activity with half of the LC_50_ value, when compared to insoluble melanin.

## 4. Conclusions

A successful production of both insoluble and soluble melanins from a single isolate of* Streptomyces* is a significant observation of the present investigation.* Streptomyces lusitanus* DMZ-3 characterized by 16S rRNA analysis was proved to be an efficient isolate for the synthesis of both melanins. An increased production (5.29 g/L) of insoluble melanin, in tyrosine medium under submerged bioprocess, was achieved by response surface methodology. The isolate had shown a greater consistency towards higher conditions of temperature (50°C) and pH (8.5) for the maximum production of insoluble melanin, using simple nutritional ingredients such as tyrosine and beef extract. An indigenous slant culture system designed and standardized for the production of pure soluble melanin from the droplets formed on the surface of the culture is a novel criterion. In an optimized slant culture system, we were able to harvest 0.264 g/L pure soluble melanin. Both melanin pigments produced by* Streptomyces lusitanus *DMZ-3 were confirmed and characterized with chemical reactions, UV, FTIR, and TLC analysis. Assessment of cytotoxicity of natural products is normally determined by either cell culture methods employing specific cell lines or using laboratory mice as experimental animals. In the present work, cytotoxicity of melanins was successfully investigated by using brine shrimps for the first time. Both insoluble and soluble melanins have been proved to be highly toxic but soluble melanin was more biologically active (0.40 *μ*g/mL) as compared to insoluble melanin (0.80 *μ*g/mL).

## Figures and Tables

**Table 1:** Optimization of critical process variables employing response surface method for the production of insoluble melanin by *Streptomyces lusitanus* DMZ-3.

Run	Critical process variables				Melanin production (g/L)	
	*X*_1_: *A*: Temperature °C	*X*_2_: *B*: pH	*X*_3_: *C*: Tyrosine %	*X*_4_: *D*: Beef extract %	Actual value	Predicted value
1	42.50	7.75	1.25	1.25	4.35	4.35
2	42.50	7.75	1.25	1.25	4.35	4.35
3	35.00	7.00	0.50	2.00	1.08	2.12
4	35.00	8.50	2.00	2.00	3.05	3.51
5	27.50	7.75	1.25	1.25	2.12	1.90
6	35.00	8.50	2.00	0.50	4.21	3.73
7	42.50	7.75	1.25	1.25	4.35	4.35
8	42.50	7.75	2.75	1.25	4.05	3.91
**9**	**50.00**	**8.50**	**2.00**	**0.50**	**5.29**	**4.65**
10	42.50	9.25	1.25	1.25	3.15	3.35
11	35.00	7.00	2.00	2.00	3.11	2.99
12	50.00	7.00	0.50	0.50	1.63	1.57
13	35.00	7.00	2.00	0.50	2.41	2.91
14	50.00	8.50	2.00	2.00	3.92	4.53
15	57.50	7.75	1.25	1.25	2.16	2.27
16	42.50	7.75	1.25	1.25	4.35	4.35
17	50.00	8.50	0.50	2.00	1.55	1.44
18	35.00	8.50	0.50	2.00	1.53	1.38
19	42.50	7.75	1.25	2.75	4.48	3.47
20	42.50	7.75	1.25	1.25	4.35	4.35
21	42.50	7.75	1.25	−0.25	2.78	3.67
22	50.00	8.50	0.50	0.50	1.89	1.72
23	35.00	8.50	0.50	0.50	1.55	1.75
24	50.00	7.00	2.00	2.00	3.20	3.40
25	42.50	7.75	−0.25	1.25	0.090	0.12
26	42.50	6.25	1.25	1.25	2.98	2.67
27	42.50	7.75	1.25	1.25	4.35	4.35
28	50.00	7.00	2.00	0.50	3.35	3.22
29	50.00	7.00	0.50	2.00	1.38	1.58
30	35.00	7.00	0.50	0.50	3.10	2.21

**Table 2:** Analysis of variance (ANOVA) of model response data.

Source	Sum of squares	Df	Mean square	*F* value	*P* value prob > *F*
Model	42.50	14	3.04	8.10	0.0001
*A*: temperature	0.21	1	0.21	0.56	0.4647
*B*: pH	0.69	1	0.69	1.84	0.1948
*C*: tyrosine	21.57	1	21.57	57.55	<0.0001
*D*: beef extract	0.061	1	0.61	0.16	0.6923
*AB*	0.38	1	0.38	1.00	0.3329
*AC*	0.90	1	0.90	2.40	0.1425
*AD*	9.506*E* − 003	1	9.506*E* − 003	0.025	0.8756
*BC*	1.61	1	1.61	4.29	0.0561
*BD*	0.086	1	0.086	0.23	0.6397
*CD*	0.026	1	0.026	0.070	0.7943
*A* ^2^	8.81	1	8.81	23.51	0.0002
*B* ^2^	3.09	1	3.09	8.24	0.0117
*C* ^2^	9.36	1	9.36	24.99	0.0002
*D* ^2^	1.04	1	1.04	2.76	0.1173
Residual	5.62	15	0.37		
Lack of fit	5.62	10	0.56		
Pure error	0.000	5	0.000		
Cor total	48.12	29			

*R* ^2^ = 0.8832					

**Table 3:** Chemical analysis of the pigments.

Color and reactions	Observations	
	Pigment 1	Pigment 2
Color	Blackish brown	Blackish brown
Solubility in water	Insoluble	Soluble
Solubility in 0.1 N NH_4_OH	Readily soluble	Readily soluble
Solubility in 0.1 N NaOH	Soluble	Soluble
Solubility in 0.1 N KOH	Soluble	Soluble
Solubility in organic solvents (Ethanol, acetone, chloroform, benzene)	Insoluble	Insoluble
Reaction with sodium dithionite and with potassium ferricyanide	Decolorized and turned to brown	Decolorized and turned to brown
Reaction with H_2_S	Reduced	Reduced

**Table 4:** Cytotoxic activity of the insoluble and soluble melanins.

Concentration of melanin (*μ*g/mL)	Log of concentration of melanin	Survived brine shrimps			Percent mortality			Lc_50_ (*μ*g/mL) at 24 h
		6 h	12 h	24 h	6 h	12 h	24 h	
Insoluble melanin								
01.0	0	19	11	06	10	45	70	0.80
02.0	0.301	17	11	07	15	45	65	
04.0	0.602	17	10	06	15	50	70	
08.0	0.903	17	09	04	15	55	80	
16.0	1.204	16	09	04	20	55	80	
32.0	1.505	16	09	03	20	55	85	
64.0	1.806	16	07	01	20	65	95	

Soluble melanin								
01.0	0	17	05	04	15	75	80	0.40
02.0	0.301	17	05	04	15	75	80	
04.0	0.602	16	05	04	20	75	80	
08.0	0.903	16	05	04	20	75	80	
16.0	1.204	15	05	03	25	75	85	
32.0	1.505	15	04	02	25	80	90	
64.0	1.806	15	03	00	25	85	100	
